# ROCK1 Is Associated with Alzheimer’s Disease-Specific Plaques, as well as Enhances Autophagosome Formation But not Autophagic Aβ Clearance

**DOI:** 10.3389/fncel.2016.00253

**Published:** 2016-11-02

**Authors:** Yong-Bo Hu, Yang Zou, Yue Huang, Yong-Fang Zhang, Guinevere F. Lourenco, Sheng-Di Chen, Glenda M. Halliday, Gang Wang, Ru-Jing Ren

**Affiliations:** ^1^Department of Neurology and Neuroscience Institute, Ruijin Hospital Affiliated to Shanghai Jiao Tong University School of MedicineShanghai, China; ^2^Neuroscience Research Australia and Faculty of Medicine, University of New South Wales (UNSW)Sydney, NSW, Australia; ^3^Research Laboratory of Cell Regulation, School of Medicine, Shanghai Jiao Tong UniversityShanghai, China

**Keywords:** Alzheimer’s disease, ROCK1, autophagy, Aβ, Beclin1

## Abstract

Alzheimer’s disease (AD) is the most prevalent form of late-life dementia in the population, characterized by amyloid plaque formation and increased tau deposition, which is modulated by Rho-associated coiled-coil kinase 1 (ROCK1). In this study, we further analyze whether ROCK1 regulates the metabolism of amyloid precursor protein (APP). We show that ROCK1 is colocalized with mature amyloid-β (Aβ) plaques in patients with AD, in that ROCK1 enhances the amyloidogenic pathway, and that ROCK1 mediated autophagy enhances the intracellular buildup of Aβ in a cell model of AD (confirmed by increased ROCK1 and decreased Beclin 1 protein levels, with neuronal autophagosome accumulation in prefrontal cortex of AD APP/PS1 mouse model). *In vitro* over-expression of ROCK1 leads to a decrease in Aβ secretion and an increase in the expression of autophagy-related molecules. ROCK1 interacts with Beclin1, an autophagy initiator, and enhances the intracellular accumulation of Aβ. Reciprocally, overexpression of APP/Aβ promotes ROCK1 expression. Our data suggest ROCK1 participates in regulating Aβ secretion, APP shedding and autophagosome accumulation, and that ROCK1, rather than other kinases, is more likely to be a targetable enzyme for AD therapy.

## Introduction

Alzheimer’s disease (AD) is the leading cause of dementia, characterized by the accumulation of extracellular and vascular amyloid in the brain. Amyloid plaques are composed of amyloid-β (Aβ) peptides, and they are derived from the sequential cleavage of the amyloid precursor protein (APP) by a set of proteases. The cleavage of APP occurs through two different pathways: a non-amyloidogenic pathway mediated by α-secretase and an amyloidogenic pathway mediated by β-secretase (Tang, 2005). α-Secretase is a metalloprotease and disintegrin enzyme that cleaves within the Aβ sequence to generate a secreted APP fragment (sAPPα). sAPPα has neurotrophic properties and promotes neurite outgrowth modulating the efficacy of synaptic neurotransmission (Ma et al., 2009). Conversely, APP cleavage by β-secretase occurs at the N-terminus with the C-terminal APP fragment subsequently cleaved by γ-secretase leading to the production of Aβ peptides.

Rho-associated coiled-coil kinases (ROCKs) are Ser/Thr kinases and the first identified effectors of Rho GTPase. They are involved in several aspects of cell behavior, such as cell motility, cell proliferation and apoptosis (Chang et al., 2006; Yang et al., 2013; Loirand, 2015; Swanger et al., 2015). Research indicates that ROCKs are potential therapeutic targets for Aβ metabolism modulation (Weggen et al., 2001; Jacobs et al., 2006; Salminen et al., 2008). Two constitutively-expressed ROCK enzymes have been identified—ROCK1 and ROCK2 that share 92% homology in their kinase domain and differ most in their regulatory domains and subcellular localization (Ishizaki et al., 1997; Shi et al., 2013; Chong et al., 2016). Recent studies show that ROCK1 modulates the shedding of the α-secretase-cleaved soluble APP ectodomain (sAPPα) from cultured cells, and that ROCK1 depletion reduces the levels of Aβ (Herskowitz et al., 2013; Henderson et al., 2016). The buildup of internal Aβ rather than its external deposition is associated with neuronal loss in APP/PS1 knock-in mice suggesting externalization of cellular Aβ is protective (Christensen et al., 2008). However, the mechanism by which ROCK1 externalizes Aβ or regulates Aβ production remains to be determined.

In AD, autophagy directly affects the secretion of Aβ and amyloid plaque formation (Nilsson et al., 2013), and long-term rapamycin treatment reduces Aβ plaque load by the induction of autophagy in an AD mouse model (Nilsson et al., 2013). The pivotal role for autophagy in the clearance of aggregate-prone proteins also manifests with an accumulation of autophagic vacuoles (autophagosomes and autolysosomes) in several neurodegenerative diseases (Zhang et al., 2016). Beclin1-mediated autophagy is the major cellular pathway for degradation of aggregated proteins and is required for the formation of double-membrane vesicles, cytoplasmic organelles called an autophagosome (McLeland et al., 2011). This dynamic process involves autophagosome formation, autophagosome-lysosome fusion and the degradation of autophagosomal contents by all kinds of hydrolases (Hong et al., 2016). During the process of autophagosome biogensis, Beclin1 plays a central role and governs the autophagic process by binding to phosphatidylinositol 3-kinase class III (PtdIns3KC3), which recruits additional autophagic proteins for autophagosome formation (Miki et al., 2016). ROCK1 phosphorylation of Beclin1 is the critical regulator for stress-induced autophagy (Gurkar et al., 2013). A reduction in ROCK1 is thought to diminish Aβ levels by enhancing lysosomal degradation of APP (Henderson et al., 2016).

To investigate the involvement of ROCK1 in the Aβ pathology diagnostic features for AD, the levels of and location of ROCK1 in brain tissue from preclinical and clinical AD patients were examined. Aβ production and autophagosome formation following increases or decreases in ROCK1 levels were tested using cell culture methods. We showed that ROCK1 is deposited with highly aggregated Aβ in human brain tissue, and that activated ROCK1 inhibits autophagic Aβ clearance via interaction with Beclin1, increasing Aβ burden during AD progression.

## Materials and Methods

### Pathological Cohort

The human ethics committee of the University of New South Wales approved this tissue study. Following institutional approvals, 10 μm formalin-fixed paraffin-embedded temporal cortex sections from 17 cases with different severities of AD pathology (female: male = 13:4, age = 87 ± 9.5 (years old), post mortem delay = 15 ± 11 (h), low severity, Braak stages 0–2 and not demented: intermediate severity and Braak stages 3–4: high severity, Braak stages 5–6 and dementia = 5:3:9) were obtained from the Sydney Brain Bank which collects brain tissue with informed consent from longitudinally followed research participants. Subjects were characterized according to recent criteria for the pathological diagnosis of AD. One of the cases with an intermediate severity had a diagnosis of AD for 3 years, while all those with a high probability of AD had dementia for between 5 and 17 years. Exclusion criteria were alternate neurodegenerative disorders or a dominant family history of neurodegenerative disorder.

### Immunofluorescence

For immunofluorescence, the 10 μm formalin-fixed, paraffin-embedded sections underwent heat-induced antigen retrieval with Citrate Buffer pH 6 for 3 min, followed by formic acid pre-treatment for 3 min. Tissue sections were double-labeled with rabbit anti-ROCK1 (1:50, Abcam) and mouse monoclonal anti-human Aβ1–42 (1:200, M0872, Dako, Glostrup, Denmark), and visualized with goat anti-rabbit Alexa Fluor 488 (1:500, A-11008, Life Technologies) and goat anti-mouse Alexa Fluor^®^ 594 (1:500, A-11005, Life Technologies), respectively. Sections were coverslipped with Vectashield HardSet Antifade Mounting Medium with DAPI (H-1500, Vector Laboratories, Burlingame, CA, USA) and examined on confocal microscope (Nikon Eclipse E400).

### Quantitative Analysis of Aβ and ROCK1 Colocalization

For quantification, 10 images were made for each case where plaques were present. All diffuse and cored plaques on each image was identified using the Aβ1–42 immunofluorescence, and the proportion that had ROCK1 co-localization recorded separately for both diffuse and core plaques. In total, an average of 40 ± 17 plaques were examined per case, with 66% on average being diffuse and 36% being cored. Multivariate statistics were performed in SPSS (IBM, v23) to assess differences between the percentages of plaques colocalizing ROCK1 immunoreactivity and the severity of cortical plaques observed, as assessed by the CERAD score (0 = none, 1 = mild or 1–5/image, 2 = moderate or 6–15/image and 3 = severe or more than 15/image), as well as to case diagnosis based on pathological load of different plaque types and neurofibrillary tangles (control, preclinical AD and AD).

### Cell Lines and Cell Culture

HEK293 cells stably transfected with human APP695 harboring the “Swedish” mutation (HEK293 APP695sw) and SH-SY5Y human neuroblastoma were maintained in Eagle’s minimal essential media or DMEM (Gibco), respectively, with 10% fetal bovine serum, and 1% penicillin/streptomycin. Cells were plated at 100,000 cells/cm^2^ density in 6-well dishes that were coated with 100 μg/ml poly-lysine. On day 2 post plating, cells were transduced with indicated lentivirus with a multiplicity of infection of 1. For all studies, cells were treated with drugs at 72 h post-transduction in conditioned media for 16 h. For RNAi knockdown of ROCK1, cells were harvested 96 h post-transduction. For transductions or transfections, equivalent amounts of cells were plated, and transfections were performed using Lipofectamine 2000 (Invitrogen) according to the manufacturer’s instructions. At 72 h post-transfection, cells were treated as indicated and then processed for western blot analysis or fluorescence imaging.

### Cell Proliferation Assay

Cell proliferation was measured by the CCK-8 Kit (Beyotime) according to the manufacturer’s instructions. Cells in 100 μl media were treated with 10 μl CCK-8 reagent at 37°C for 2 h, followed by measuring the absorbance at 450 nm on a microplate reader.

### Plasmid Construct, RNA Interface and Transfection

ROCK1 plasmid (pCAG-myc-ROCK1^myc-727^ Δ3, ROCK1 CA) was constructed as previously reported (Ishizaki et al., 1997). The siRNA sequence against ROCK1 (sense 5′-GGCAGAGGAAGAAUAUAAATT-3′; antisense 5′-UUUAUAUUCUUCCUCUGCCTT-3′) and scrambled siRNA was synthesized by GenePharma (Shanghai, China). Cells were transfected with ROCK1 plasmid, ROCK1 siRNA or scrambled siRNA using Lipofectamine 2000 reagent according to the manufacturer’s instructions. At 72 h post-transfection, cells and supernatant were harvested and then processed for western blot analysis.

### Aβ Measurements

For all cells, conditioned media were used for 48 h, and then the cells and media collected separately for biochemical analyses. Aβ40 was detected using a sandwich ELISA for human Aβ40 following the manufacturer’s instructions. Plates were read at 450 nm on a Synergy MX plate reader (BioTeck, Winooski, VT, USA).

### Immunoblotting

Cells were lysed with 1% RIPA Lysis Buffer (Beyotime, China). Cell lysate was subjected to a 13,000 rpm spin to remove nuclei and debris. Cleared lysate was used for the indicated biochemical assay. Protein concentrations were determined using the Enhanced BCA Protein Assay Reagent (Beyotime). Equal amounts of cell lysate were loaded onto SDS-PAGE gels and then transferred to PVDF membranes. Membranes were blocked with 5% fat-free dry milk in Tris buffered saline (TBS), containing 0.05% Tween-20, and incubated with primary antibodies. Protein bands were detected by horseradish peroxidase-conjugated species-specific secondary antibodies. Actin was used as loading control. Images were captured using an Odyssey Image Station (LI-COR), and band intensities were quantified using Scion Image.

### mCherry-GFP-LC3B Assay

In order to evaluate the effects of ROCK1 plasmid on GFP–LC3B puncta formation, SH-SY5Y cells were plated on poly-lysine-coated 96-well plates and transduced with adenovirus expressing mCherry-GFP-LC3B fusion protein. After 24 h and 48 h, cell images were obtained by EVOS fl Auto (Life, USA).

#### Co-Immunoprecipitation

Co-immunoprecipitation (CoIP) was performed as previously described (Rijal Upadhaya et al., 2014). HEK293 cells in 60-mm dishes were co-transfected with pCAG-myc-ROCK1^myc-727^ Δ3 plasmid. Forty eight hours later, the cells were lysed with 1% RIPA Lysis Buffer (Beyotime). After clarification by centrifugation at 4°C for 30 min at 12,000 rpm, 500 μl cell lysates were incubated with 20 μl of protein A + G agarose beads (Beyotime, China) for 4 h with gentle rotation at 4°C. The beads were washed four times with the cell lysis buffer and precipitates were eluted with 2× SDS-PAGE sample buffer, and then analyzed by western blot for anti-Myc and anti-Beclin1 immunoreactivity, respectively.

#### Primary Neuron Culture, Transfection and Immunofluorescence

Primary cortical neurons were prepared from E16 to E18 days of Sprague-Dawley rat brain tissues. Briefly, cerebral cortex was removed aseptically, and then digested and dispersed into single cells; then neurons were re-suspended in neuro-basal medium with 2% B27 supplement (Invitrogen), 2 mM glutamine and 1% penicillin and streptomycin (Invitrogen, Carlsbad, CA, USA) and then seeded at 1.25 × 10^5^ cells per cm^2^ on 12-well plates (Corning Inc., Midland, NC, USA) coated with 100 μg/ml poly-l-lysine and incubated in humidified atmosphere with 5% CO_2_ at 37 °C. Transfection using Lipofectamine 2000 (Invitrogen) was carried out on acutely dissociated neurons before plating, and 4 h later, the whole medium was replaced with NB/B27 and immunofluorescence experiment was performed after 48 h transfection.

For immunofluorescence, primary neurons were cultured on a cover glass and fixed with 4% paraformaldehyde in PBS for 10 min at room temperature. Permeabilization was performed in PBS with 0.3% Triton X-100 for 10 min at room temperature. After blocking for 30 min with 5% normal donkey serum, neurons were incubated with rabbit anti-LC3 (1:100, CST) antibody or mouse anti-ROCK1 (1:500, Abcam) antibody overnight at 4°C. After washing three times with 0.1 M PBS, the sections were incubated with AlexaFluor 488-conjugated donkey anti-rabbit or anti-mouse IgG secondary antibodies (Invitrogen), respectively, and the sections were visualized with a confocal microscope (FV-1000).

#### Immunohistochemistry and Histology

APP/PS1 mice were provided by Model Animal Research Center of Nanjing University. APP/PS1 mice have been intensively characterized for Aβ plaques load and AD behavioral phenotypes. In this experiment, 10-month-old APP/PS1 transgenic mice and age-matched C57BL/6J were used. Mice were deeply anesthetized and perfused with 10% formalin, and their brains were removed and fixed in 4% paraformaldehyde in PBS overnight at 4°C. Whole brains were cryoprotected in 25% sucrose in 0.1M PBS at 4°C until sectioning. The brain tissues were cut in to coronal 30 μm sections. Immunohistochemistry (IHC) for APP and Beclin1 were performed using primary antibodies of 6E10 (1:100, Novus) and rabbit anti-Beclin1 (1:100, Novus) respectively (Sugrue et al., 2010). APP and Beclin1 immunopositive neurons were quantified according to the previously established method (Dlugos and Pentney, 2002).

#### Antibodies and Reagents

Mouse anti-ROCK1 antibody (Abcam, 1:500 dilution), rabbit anti-Beclin1 (Novus, 1:10,000), rabbit anti-APP-CTF (Sigma-Aldrich, 1:5000), anti-mouse sAPPα (IBL, 1:50), anti-mouse myc (Abcam, 1:1000), anti-rabbit LC3 (Cell Signaling, 1:100) and mouse anti-β-actin (Sigma-Aldrich, 1:1000) were used. Loading controls (β-actin) were used for Western blot standardization. Lipofectamine 2000 was sourced from Invitrogen. 3-methyladenine (3-MA) was purchased from Sigma Aldrich (St. Louis, MO, USA) and 3-MA was dissolved in double distilled water and titrated to 1 mM.

#### Statistical Analysis of the Cell Culture Experiments

Data are reported as mean ± SEM. Comparisons between experimental groups were analyzed by Student’s *t*-test or ANOVA as appropriate, with *p* < 0.05 considered as significant.

## Results

### ROCK1 is Associated with Aβ Plaques in the Brains of AD Patients

**Figure 1:**
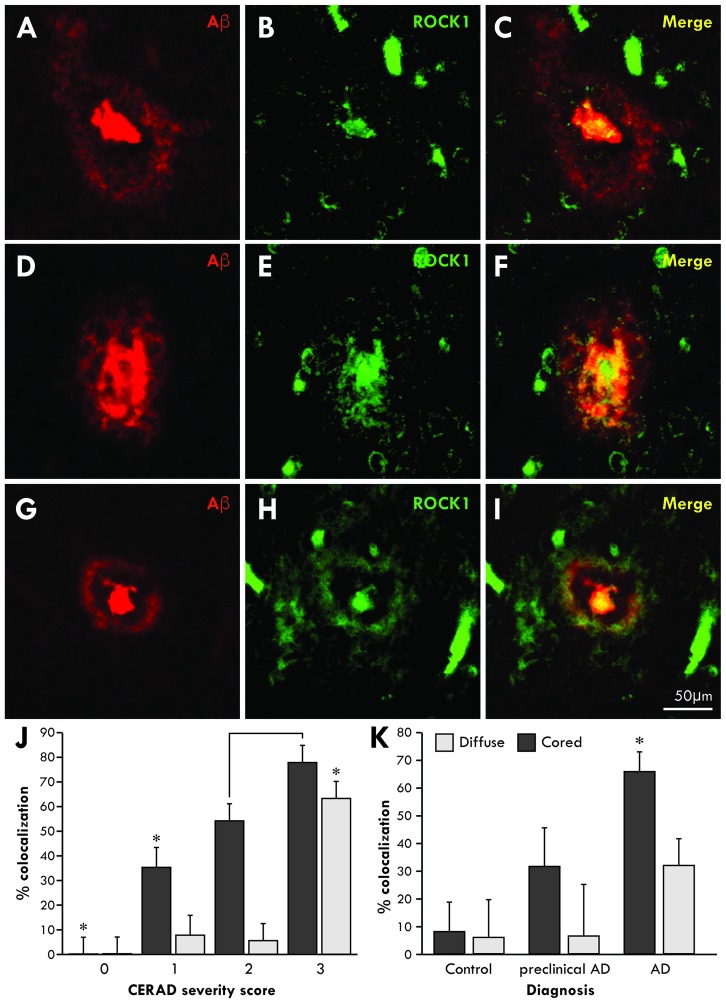
**Co-localization of Rho-associated coiled-coil kinase 1 (ROCK1) and amyloid-β (Aβ) in cortical plaques.** Confocal microscopy using specific antibodies to Aβ (red) and ROCK1 (green) revealed a high degree of co-localization in the dense cores of cortical Aβ plaques and less overlap with diffuse Aβ depositions in the cortex **(A–I)**. Quantitation of the degree of colocalization in Aβ plaques was assessed in 40 ± 17 temporal lobe plaques in each case with Aβ plaque deposition (*n* = 14/17). Multivariate analysis identified a significant increase (*p < 0.02) in ROCK1 and Aβ colocalization in plaques with increasing cortical deposition of Aβ plaques, as assessed by the CERAD plaque score **(J)**. With increasing CERAD plaque severity, more cored plaques contained ROCK1 immunoreactivity, whereas colocalization of ROCK1 and Aβ in diffuse plaques was mainly observed when there was a high cortical load of Aβ **(J)**. Multivariate analysis revealed that the degree of colocalization of ROCK1 and Aβ in cored plaques was highest in patients with clinical Alzheimer’s disease (AD; *p = 0.003), although significant numbers of Aβ plaques contained ROCK1 immunoreactivity in preclinical AD **(K)**.

To determine whether Aβ associates with ROCK1 in the brains of patients with AD, co-localization studies were performed using specific antibodies in healthy aged controls (*n* = 5 aged 89 ± 4 years, Braak neuritic stages 0–2), preclinical AD (*n* = 4 aged 85 ± 3 years, Braak neuritic stages 3–4) and clinical end-stage AD (*n* = 8 aged 86 ± 4 years, Braak neuritic stages 5–6). Consistent with this finding, ROCK1 immunoreactivity was identified in more cored than diffuse plaques (Figure 1). Quantitation showed that the majority of cored plaques contained ROCK1 immunoreactivity, and that colocalization increased with increasing severity of cortical Aβ deposition as assessed by CERAD plaque score (Figure 1J, Wilks’ Lambda *p* < 0.0001 covarying for age and brain size, *post hoc* Bonferroni *p* = < 0.002). Nearly half of all Aβ plaques in preclinical AD contained ROCK1 immunoreactivity, with significantly higher numbers of ROCK1-immunopositive Aβ plaques found in AD patients (Figure 1K, Wilks’ Lambda *p* = 0.03 covarying for age and brain size, *post hoc* Bonferroni *p* = 0.003 for AD diagnosis). These results demonstrate a close relationship between ROCK1 localization and pathological Aβ plaque in both preclinical and clinical AD.

### ROCK1 Regulates Aβ Secretion

**Figure 2:**
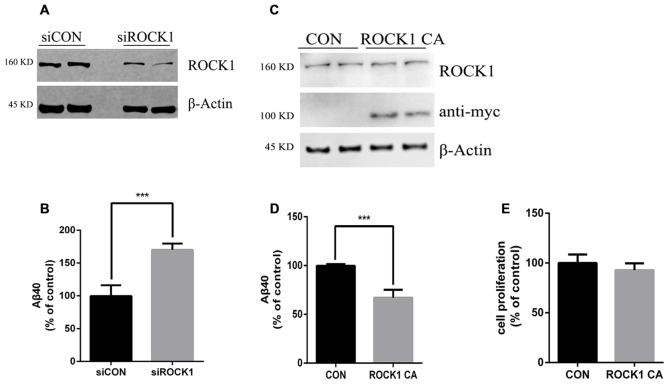
**ROCK1 levels modulate Aβ secretion. (A,C)** Effects of knocking down ROCK1 levels **(A)** and increasing constitutively activated ROCK1 (ROCK1 CA) levels **(C)** on secreted Aβ. HEK293 cells were transfected with ROCK1 siRNA (siROCK1), scramble siRNA (siCON), ROCK1 CA plasmid and pcDNA (Con) and 48 h post-transfection secreted Aβ40 was measured by ELISA. **(B,D)** Representative immunoblot confirms ROCK1 knockdown **(B)** and increase **(D)**. **(E)** Cell proliferation assay following ROCK1 CA plasmid transfection. Data are presented as Mean ± SEM. Graphs represent % of control groups, *n* = 10; students’ *t* test, ***p < 0.01.

To further explore the effects of ROCK1 cleavage on the production of Aβ, we constructed a plasmid expressing this constitutively active ROCK1 fragment (ROCK1 CA), as previously reported (Ishizaki et al., 1997). In HEK 293T cells stably transfected with the APP695 Swedish mutation (APPsw), ROCK1 levels were manipulated by transfection with ROCK1 CA plasmid, ROCK1 siRNA or scrambled siRNA. Forty eight hours after transfections, the levels of secreted Aβ40 in the cell culture medium were measured by ELISA. As shown in Figure 2, secreted Aβ40 levels were increased by 80% compared to the control after depletion of ROCK1 (Figures 2A,B), which is consistent with a recent study. In contrast, secreted Aβ40 levels were decreased following ROCK1 CA over-expression in HEK293T APPsw cells (Figures 2C,D). The Aβ42 level was too low to be detected by ELISA. ROCK1 CA plasmid transfection did not change cell proliferation (Figure 2E). These results confirm that ROCK1 influences Aβ secretion.

### ROCK1 Modulates APP Shedding

**Figure 3:**
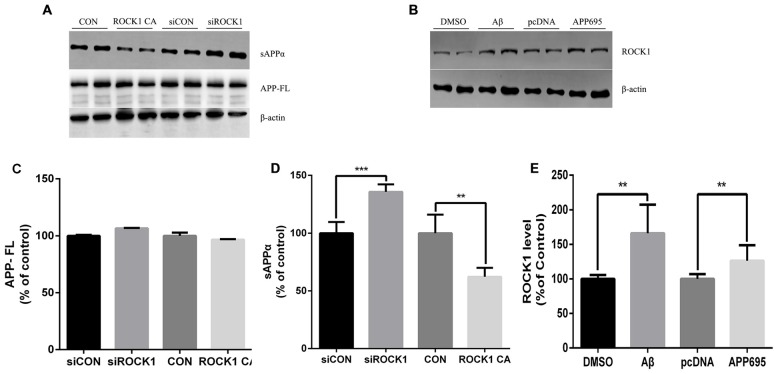
**ROCK1 modulates amyloid precursor protein (APP) shedding. (A)** Immunoblot analysis of sAPPα and APP full-length (APP FL) following ROCK1 knockdown and ROCK1 increase. **(B)** Immunoblot of ROCK1 after Aβ40 treatment and APP695 plasmid transfection in HEK 293T cells. ** (C–E)** Quantification of APP FL **(C)**, sAPPα **(D)** and ROCK1. Data are presented as Mean ± SEM, *n* = 12, students’ *t* test, ***p < 0.001, **p < 0.01.

Given that there is an established relationship between ROCK1 and Aβ secretion, we next investigated the effects of ROCK1 on APP cleavage. After transfection of ROCK1-targeted siRNA or ROCK1 CA plasmid to HEK293T APPsw cells, cells were harvested 48 h later and lysates were analyzed. The holo-APP and sAPPα were examined by Western Blot with the mAb 6E10, specific for residues 1–17 of the Aβ sequence (Figure 3A). Electrophoresis revealed no significant difference of full-length APP (Figure 3C), however the amount of sAPPα was higher after ROCK1 depletion (Figure 3D). In cells with increased ROCK1 CA levels, sAPPα was significantly decreased (Figure 3D). These results confirm that ROCK1 modulates the metabolism of APP. To further explore the interaction between ROCK1 and APP/Aβ, we performed normal HEK293T cell culture with Aβ40 treatment or APP695 plasmid transfection. Addition of Aβ40 or APP695 over-expression promoted endogenous ROCK1 expression in normal HEK293T cells (Figures 3B,E).

### Identification of ROCK1 as an Enhancer of Beclin1 Dependent Autophagy

**Figure 4:**
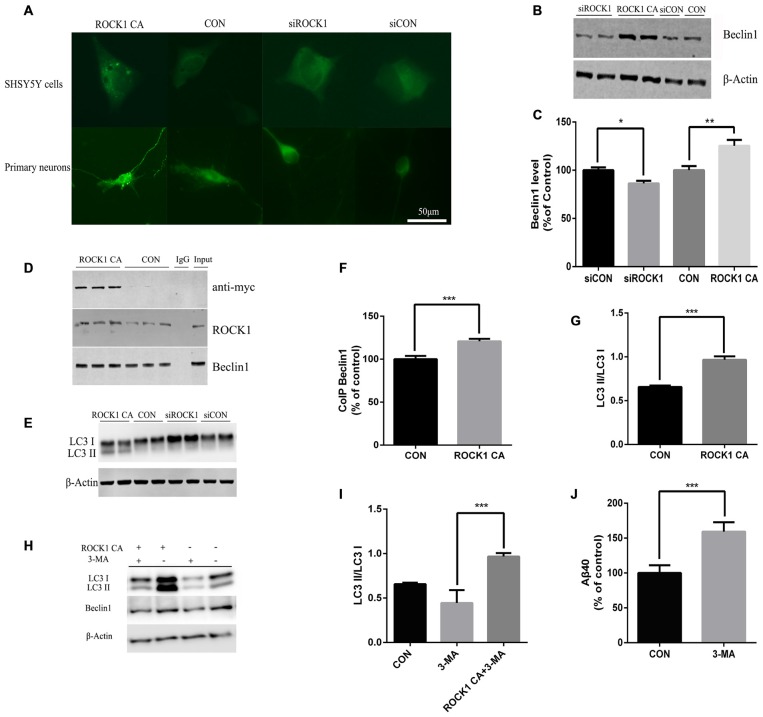
**ROCK1 increases autophagy via an interaction with Beclin1. (A)** Increased ROCK1 CA levels induced an increase in the intracellular autophagosome formation and accumulation. SH-SY5Y cells (upper panels) and primary neurons (lower panels) were co-transfected with ROCK1 CA plasmid and mCherry-GFP-LC3B adenovirus (pcDNA was used as a control). Fluorescence microscopy was used to detect the formation of GFP-LC3 puncta after 24 h post-transfection. **(B)** Expression of Beclin1 following ROCK1 knockdown and ROCK1 CA over-expression. **(C)** Quantification of Beclin1 following ROCK1 knockdown and ROCK1 CA over-expression. **(D)** HEK293 cells transfected with ROCK1 CA plasmid and pcDNA. Forty eight hours post-transfection co-immunoprecipitation (CoIP) was performed with cell lysates by using ROCK1 crosslinked agarose and then blotted with antibodies. **(E)** Representative immunoblot of LC3 II/LC3 I after ROCK1 CA over-expression and ROCK1 knockdown. **(F)** Quantification of CoIP Beclin1 levels. **(G)** Representative immunoblot of LC3 II/LC3 I after ROCK1 CA over-expression and ROCK1 knockdown. **(H)** Representative immunoblot of LC3 II/LC3 I after ROCK1 CA over-expression and ROCK1 knockdown. **(I)** ROCK1 CA plasmid transfection reverses 3-methyladenine (3-MA) induced autophagy inhibition. Representative immunoblot of LC3 II/LC3 I. **(J)** Increased Aβ40 secretion after 3-MA treatment (48 h) by ELISA. Data are presented as Mean ± SEM, students’ *t* test, *p < 0.05, **p < 0.01, ***p < 0.001.

In further experiments, we explored the mechanism of ROCK1 as an enhancer of Beclin 1 dependent autophagy. When ROCK1 was depleted, the expression of Beclin1 was decreased, and when ROCK1 CA was over-expressed by plasmid transfection, the level of Beclin1 was also increased. To determine whether autophagy is involved in the mechanism by which ROCK1 depletion increases Aβ secretion, SH-SY5Y cells were co-transfected with mCherry-LC3B adenovirus and ROCK1 CA plasmid and the patterns of LC3 puncta were analyzed by immunoflouresence. Figure 4A shows an increase in LC3 puncta after ROCK1 CA plasmid transfection. LC3 was also assessed in HEK293T cells and, as the results show, the LC3II/I ratio was increased in cells when ROCK1 CA was over-expressed and decreased following ROCK1 depletion (Figures 4E,G). We also explored whether ROCK1 interacted with Beclin1 during autophagy (Figures 4B,C). HEK293T cells transfected with myc-tagged human ROCK1 were lysed and the proteins subjected to co-immunoprecipitation with anti-ROCK1 and anti-myc antibody. Our results showed that endogenous ROCK1 and Beclin1 interacted with each other. Moreover, when ROCK1 CA was over-expressed, this interaction was enhanced (Figures 4D,F). These results showed that an interaction between ROCK1 and Beclin1 triggered the autophagic build-up of intracellular Aβ and a decrease in Aβ secretion.

To further elucidate ROCK1 influence on autophagy, 3-MA was used as an inhibitor of autophagy. After HEK293T APPsw cells were treated with 3-MA for 6 h, they were transfected with ROCK1 CA plasmid for 48 h. To test whether ROCK1 CA over-expression rescued 3-MA-induced autophagy inhibition, the cells were lysed and proteins analyzed by ELISA and Western blot. Inhibiting autophagy increased intracellular Aβ40 levels (Figure 4J), and over-expression of ROCK1 by transfection with ROCK1 CA plasmid reversed this effect even though 3-MA treatment decreased Beclin1 levels (Figures 4H,I).

### APP/PS1 Mice have Increased ROCK1 and Neuronal Autophagosome Accumulation

**Figure 5:**
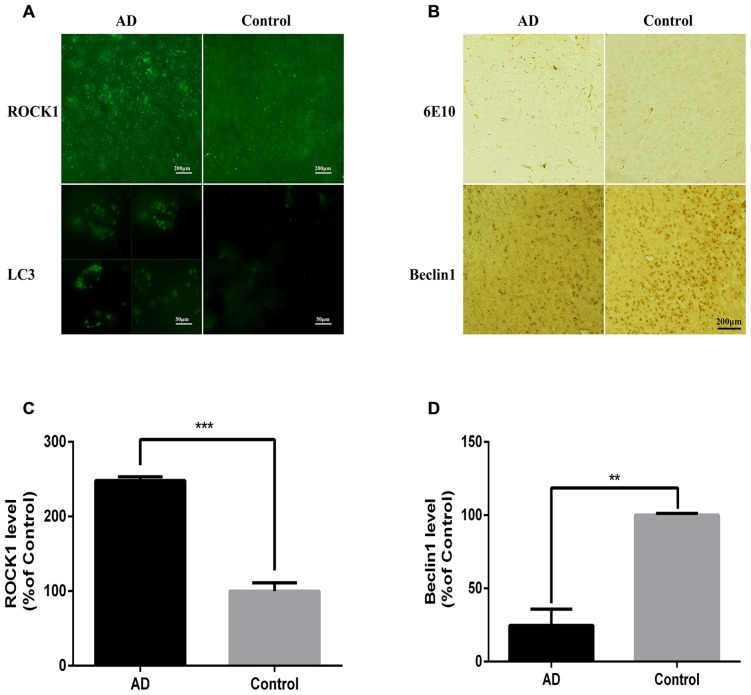
**ROCK1 is up-regulated with neuronal autophagosome accumulation in AD mice. (A)** Representative ROCK1 (upper panels) and LC3 (lower panels) immunofluorescence in AD and normal mouse brain sections. **(B)** Representative immunohistochemical (IHC) micrographs of Aβ (6E10, upper panels) and Beclin1 (lower panels) immunofluorescence in AD and normal mouse brain sections. **(C)** Quantification of ROCK1 immunopositive neurons in AD mouse brain sections compared to control sections (*n* = 4). **(D)** Quantification of Beclin1 immunopositive neurons in AD mouse brain sections compared to control sections (*n* = 4). Data are expressed as mean ± SEM; **p < 0.01, ***p < 0.001.

After confirming the relationship between ROCK1 and autophagosome formation *in vitro*, we next assessed whether ROCK1 and autophagosome proteins are upregulated with Aβ *in vivo* by conducting immunofluorescence and IHC staining of brain sections from 10 months old APP/PS1 transgenic C57BL/6 mice. Our data showed that ROCK1 was increased significantly with neuronal autophagosomes accumulation (Figures 5A,C) and that Beclin1 was decreased in the AD mouse model (Figures 5B,D). These observations suggested that autophagy was impaired in neurons and increased ROCK1 inhibited Aβ secretion, resulting in intracellular Aβ accumulation and neurotoxicity. Taken together, these data demonstrated the same associations between ROCK1, intracellular Aβ and autophagosomes accumulation as we identified in our *in vitro* experiments.

## Discussion

The critical events that trigger or cause increased Aβ levels and post-translational modifications that increase Aβ insolubility are still under debate. However, modulation of the production, metabolism and fibrilization of the protein are key pathogenic aspects. Our results show that ROCK1 levels regulate Aβ clearance by modulating the autophagy pathway via an interaction with its substrate, Beclin1, that initiates autophagosome formation that then accumulate intracellularly. This is consistent with previous studies showing that ROCK1 regulates autophagosome formation (Gurkar et al., 2013), that autophagosomes accumulate in AD and that ROCK1 inhibition reduces Aβ levels in brain (Nixon and Yang, 2011). Our data also show that ROCK1 collocates with fibrilized Aβ. It has recently been shown that different post-translational modifications of Aβ occur in different types of plaques (Rijal Upadhaya et al., 2014). The cases in this study have been previously analyzed using Western blotting for ROCK1 with no reduction in membrane associated SDS-soluble ROCK1 protein levels by end-stage AD (Wang et al., 2016). Taken together, our data indicate that ROCK1 may be involved in Aβ modification.

Phosphorylation of Aβ at serine 8 at the cell surface has recently been identified to dramatically increase the rate and amount of Aβ oligomerization and amyloid fibril formation (Rezaei-Ghaleh et al., 2016). In fact, AD patients are distinguished from those without clinical AD by selectively accumulating this phosphorylated form of Aβ (Rijal Upadhaya et al., 2014). The kinases that are involved in phosphorylating Aβ are poorly understood, but are thought to be of the AGC kinase family of serine-threonine kinases (Rezaei-Ghaleh et al., 2016), which includes ROCK1. Unlike ROCK2, ROCK1 interacts with a number of proteins in the plasma membrane (Stroeken et al., 2006) and we show that ROCK1 co-localizes with highly fibrilized Aβ in human brain. The localization and association of ROCK1-associated Aβ with clinical AD mirrors the data observed for serine 8 phosporylated Aβ (Rijal Upadhaya et al., 2014) and suggests that ROCK1 may be the kinase involved in this disease-specific Aβ phosphorylation.

We provide further evidence that ROCK1 has a role in the plasma membrane in its influence on APP shedding. APP shedding is directly mediated by α- and β-secretase generating either non-amyloidogenic (sAPPα) or amyloidogenic fragments (Tang, 2005). Our data show that ROCK1 activation enhances the amyloidogenic pathway, consistent with recent data (Henderson et al., 2016). However, we demonstrate that ROCK1 decreased sAPPα shedding in cultured cells, but also reduced rather than increased Aβ secretion from the cells, suggesting that ROCK1 may also play a role in Aβ secretion at the plasma membrane (potentially associated with the aberrant phosphorylation of Aβ, see above). Together these results are also consistent with ROCK1 activation increasing intracellular Aβ.

We show that activated ROCK1 inhibits the autophagic clearance of Aβ via its interaction with Beclin1, increasing Aβ burden during AD progression. Autophagy is a critical clearance system for cellular toxic protein aggregates and it is crucial for the physiological regulation of normal cell function, which can be induced by extracellular and intracellular signals, including oxidative stress, starvation and endoplasmic reticulum stress (Wang and Mao, 2014). In the brain, the complex axonal and dendritic structures are highly dependent on efficient autophagic turnover (Wong and Holzbaur, 2015; Hong et al., 2016). In AD, autophagy is upregulated at early disease stages, as evidenced by swollen endosomes and the accumulation of autophagosomes (Wong and Holzbaur, 2015). At the transcriptional level, autophagy-activating factors are also upregulated in AD (Jiang et al., 2013). ROCK1 phosphorylation of Beclin1 is known to be the critical regulator for stress-induced autophagy (Gurkar et al., 2013). We confirm that Beclin1 is a substrate of ROCK1 and that the interaction of ROCK1 and Beclin1 results in the inhibition of autophagic Aβ clearance. It is well known that Beclin1 is a critical regulator of autophagy and several lines of investigation have demonstrated that autophagy plays an important role in Aβ clearance (also known as “autophagic Aβ clearance”; Wirawan et al., 2012). Although ROCK1 phosphorylation of Beclin1 activates autophagosome production, our data show that these organelles accumulate intracellularly with enhanced intracellular Aβ and a reduction in secreted Aβ. This is consistent with previous reports showing that autophagosome maturation to autolysosomes is the main problem with autophagy in AD and results in accumulating intracellular Aβ (Nixon and Yang, 2011).

Overall our data suggest a role for ROCK1 in regulating Aβ production, secretion and post-translational modification at the plasma membrane, as well as its important role in intracellular autophagosome induction. The location of ROCK1 to the plasma membrane (Stroeken et al., 2006), and also in extracellular plaques (as shown in this study), makes this kinase potentially more easily targetable and relevant for therapeutic manipulations than others. If as suggested, Aβ phosphorylation at serine 8 enhances plaque maturation to progress presymptomatic pathology to symptomatic AD (Rijal Upadhaya et al., 2014), then inhibition of ROCK1 and/or Aβ dephosphorylating enzymes, may provide the most relevant treatments. Taken together, our findings outline a critical role of ROCK1 in the progression of Aβ pathology, highlighting its potential as a therapeutic target.

## Author Contributions

Y-BH and YZ performed most experiments, collected and analyzed data, and wrote the manuscript. YH and GFL performed pathological experiments. GW, R-JR, S-DC and GMH participated in the design of the study. Manuscript was written by Y-BH and YH, and critically reviewed by GW, R-JR, Y-FZ, S-DC and GMH. All authors read and approved the final manuscript.

## Conflict of Interest Statement

The authors declare that the research was conducted in the absence of any commercial or financial relationships that could be construed as a potential conflict of interest.

## Abbreviations

AD: Alzheimer’s disease
APP: amyloid precursor protein
Aβ: amyloid-β
CoIP: co-immunoprecipitation
PtdIns3KC3: phosphatidylinositol 3-kinase class III
ROCK1: Rho-associated coiled-coil kinase 1
3-MA: 3-methyladenine.
